# Deciphering Ultra-High Dose Rate Irradiation with *Drosophila melanogaster*

**DOI:** 10.3390/antiox15060736

**Published:** 2026-06-10

**Authors:** Marvin Kreuzer, Irene Vetrugno, Riccardo Dal Bello, Stephanie Tanadini-Lang, Erich Brunner, Darlina von Salis, Damian Manetsch, Sandipan Tewary, Matthias Guckenberger, Jamie Little, Martin Pruschy

**Affiliations:** 1 Department of Radiation Oncology, University Hospital Zurich and University of Zurich, 8091 Zurich, Switzerland; 2 Institute of Molecular Life Sciences, University of Zurich, 8057 Zurich, Switzerland

**Keywords:** UHDR irradiation, FLASH, *Drosophila melanogaster*, lifespan, geotaxis, ferroptosis

## Abstract

FLASH RT, which employs ultra-high dose rates (UHDR), has shown potential in reducing irradiation-induced damage to normal tissue while maintaining effective tumor targeting. For successful clinical translation, mechanistic explanation behind the so-called FLASH effect has yet to be deciphered and new in vivo model systems for mechanistic studies are therefore of high demand. We investigated the differential effects of UHDR (3000–7000 Gy/s) and conventional (CONV) (0.5 Gy/s) irradiation in *D. melanogaster* by irradiating adult female flies with 16 MeV and 9 MeV electron beams using an adapted clinical linear accelerator. Substantial lifespan prolongations were observed in single high-dose UHDR-irradiated groups compared to CONV irradiation groups with increasing doses (1000–1500 Gy). Split-dose UHDR irradiation further increased the lifespan compared to single high-dose UHDR irradiation. In addition, climbing deficits were induced by 300 Gy of CONV irradiation, but not by single high-dose UHDR irradiation. Additionally, we identified increased levels of lipid peroxidation in *D. melanogaster* brains indicating ferroptosis following CONV irradiation, which was not observed after single high-dose UHDR irradiation. Using relevant biological endpoints, we here demonstrate *D. melanogaster* with its advantageous characteristics to be a highly practical preclinical model organism to mechanistically investigate differential responses to UHDR and CONV irradiation.

## 1. Introduction

The goal of radiotherapy (RT) is to induce cytotoxic DNA damage in tumor cells while sparing irradiated normal tissues from damage caused by ionizing radiation. Ultra-high dose rate (UHDR) RT delivers ionizing radiation at average dose rates ≥ 40 Gy/s and has recently emerged as a potential advancement in the RT field to widen the therapeutic window. Ultra-fast radiation delivery not only dramatically shortens treatment times but can also trigger a unique biological response. The FLASH effect refers to the observed ability of UHDR-RT to reduce normal tissue damage while maintaining robust tumor cell damage and tumor control [1]. To date, the delivery of UHDR-RT with electrons and protons has been tested on different preclinical models, which provided mechanistic and efficacy-oriented insights into the potential benefits and limitations of this approach [2,3,4,5,6,7,8]. However, non-confirmatory results of the FLASH effect have also been observed when using UHDR irradiation on rodents [9], zebrafish [10], or domestic animals [11,12]. With a few clinical trials ongoing, UHDR-RT has been described in the clinics on one case of T-cell cutaneous lymphoma, where the treatment was found to be feasible and safe, yielding a positive outcome for both normal tissue toxicity and tumor control [13,14]. Nevertheless, the appropriate radiation delivery with regard to dose per fraction, instantaneous and average dose rates to apply the FLASH effect in the clinics is still much disputed. Despite UHDR-RT being recognized as an important opportunity in the RT field for minimizing normal tissue toxicity while maintaining antitumor efficacy, a mechanistic explanation behind the FLASH effect has yet to be deciphered. Even though several hypotheses have been formulated to explain the FLASH effect, e.g., self-detoxification of reactive oxygen species generated by ionizing radiation, differential lipid peroxidation and mitochondrial metabolism in normal tissues ([15,16] and refs therein), deeper mechanistic insights are needed to define the conditions for UHDR-RT applications in the clinic.

*Drosophila melanogaster* (*D. melanogaster*), commonly known as the fruit fly, has been used for decades as a model organism to investigate both immediate and long-term responses to ionizing radiation, including DNA damage, cellular apoptosis, regenerative mechanisms, immune responses and the identification of modulators involved in irradiation responses [17]. The advantageous characteristics of *D. melanogaster*, such as fast generation time and significant biological parallels to higher organisms, small size, easy maintenance and the rapid generation of genetically engineered organisms, make *D. melanogaster*, despite its high radiation tolerance, an attractive model to study the biological processes of UHDR-RT [18,19,20,21,22]. Only one study has so far been documented with *D. melanogaster*, investigating the response to low-energy X-rays using conventional and ultra-high dose rates in third instar larvae [23]. To the best of the authors’ knowledge, no prior studies have been documented with adult *D. melanogaster* flies as a more robust in vivo model in the context of UHDR-RT. However, the identification of the FLASH effect in the adult fruit fly is a prerequisite for exploiting its advantages.

In the current study, we use a previously UHDR-converted electron linear accelerator to perform both UHDR and conventional dose rate (CONV) irradiation [24]. Using two relevant biological endpoints, we show here that *D. melanogaster* responds differentially to UHDR and CONV radiotherapy, making it a practical preclinical model organism for investigating the mechanism of action of UHDR-RT. Additionally, we provide cell death-related mechanistic insights into the distinct biological effects of UHDR and CONV irradiation.

## 2. Materials and Methods

### 2.1. D. melanogaster Maintenance

The *D. melanogaster* strain *yellow*–, *white*–(*yw*;+;+) was provided by Konrad Basler (Department of Molecular Life Sciences, University of Zurich). Flies were kept at 25 °C under a 12:12 h light/dark cycle on standard cornmeal medium, prepared per liter with the following components: 100 g fresh yeast, 55 g cornmeal powder, 10 g organic wheat flour, 8 g agar, 75 g white sugar and 15 mL nipagin. Mated females aged 2–5 days were used for the experiments. According to the Cantonal Veterinary Office Zurich, experiments with *D. melanogaster* as performed in this manuscript do not require approval by the Cantonal Veterinary Office.

### 2.2. Irradiation of D. melanogaster with a Clinical Linear Accelerator and Dosimetry

**Figure A1:**
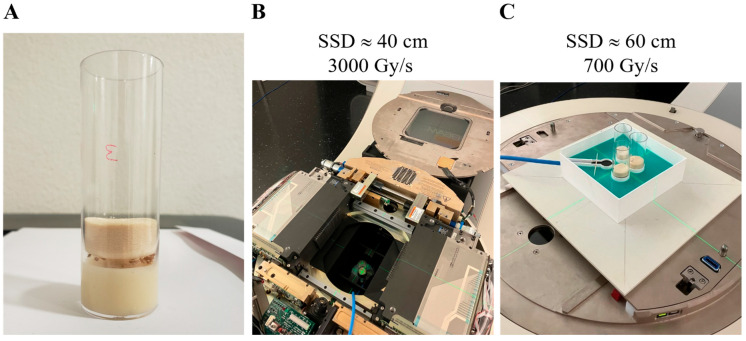
Experimental setups for irradiating adult *D. melanogaster* flies with a clinical linear accelerator. (**A**) Standard *D. melanogaster* food vial with the foam lid lowered to approximately 5 mm above the food for irradiation. (**B**) Irradiation setup for lifespan assays at SSD ~40 cm, using up to 1500 Gy of UHDR irradiation with an average dose rate of 3000 Gy. (**C**) Irradiation setup for negative geotaxis assays at SSD ~60 cm, using up to 300 Gy of UHDR irradiation with an average dose rate of 700 Gy/s.

Irradiation was performed using a converted TrueBeam linear accelerator (Varian Medical Systems, Palo Alto, CA, USA ), which was used as a research platform to deliver 9 MeV and 16 MeV UHDR electron beams, as described in [24]. Vials containing food of 1 cm (9 MeV experiments) or 2 cm (16 MeV experiments) height were filled with approximately 20 flies and sealed with foam plugs, which were lowered to approximately 5 mm above the food prior to irradiation. The vials were then placed into custom-made 3D-printed phantoms [24]. The phantoms were optimized to ensure a unform dose in the volume 5 mm above the food where the Drosophila were confined and then a detailed dosimetric characterization was performed [25]. Different irradiation setups were used depending on the required total dose. This was necessary due to the linac safety design limiting UHDR to deliveries of up to 99 pulses in one beam-on session. For lifespan assays requiring doses up to 1500 Gy, the UHDR irradiations were performed with a phantom containing a single vial positioned at a source-to-surface distance (SSD) of approximately 40 cm with an average and instantaneous dose rate of approximately 3000 Gy/s and approximately 4 × 10^6^ Gy/s, respectively (see also Figure A1 for set-up and Supplementary Tables S1 and S2). For the negative geotaxis assay, 300 Gy of UHDR irradiation were delivered using a phantom designed for the simultaneous irradiation of three vials. The phantom was placed at the interface mount with an SSD of approximately 60 cm, and an average and instantaneous dose rate of approximately 700 Gy/s and approximately 7.1 × 10^5^ Gy/s, respectively, was applied. Finally, CONV (average 0.5 Gy/s) irradiation was performed at the interface mount (SSD = 60 cm) with a phantom that allowed the simultaneous irradiation of ten vials [25]. Split-dose UHDR irradiations were also investigated by dividing the dose into five beam-on sessions with a waiting time of 30 s between each beam-on. In lifespan experiments comparing single high-dose UHDR and CONV irradiation, 16 MeV electron beams were applied. For comparisons between split dose and single high-dose UHDR irradiation, as well as in the negative geotaxis assay, 9 MeV electron beams were used.

### 2.3. Lifespan Assay

Flies were subjected to CONV and single high-dose UHDR irradiation with doses of 500, 750, 1000, 1250, and 1500 Gy in a single fraction using 16 MeV electron beams. For the comparison of single high-dose and split-dose UHDR irradiation, the flies were exposed to 1250 Gy using 9 MeV electron beams. The number of dead flies was recorded daily, and surviving flies were transferred into vials with fresh food every two days until all irradiated flies had died. Flies that did not respond to gentle prodding were scored as dead.

### 2.4. Negative Geotaxis Assay

**Figure A2:**
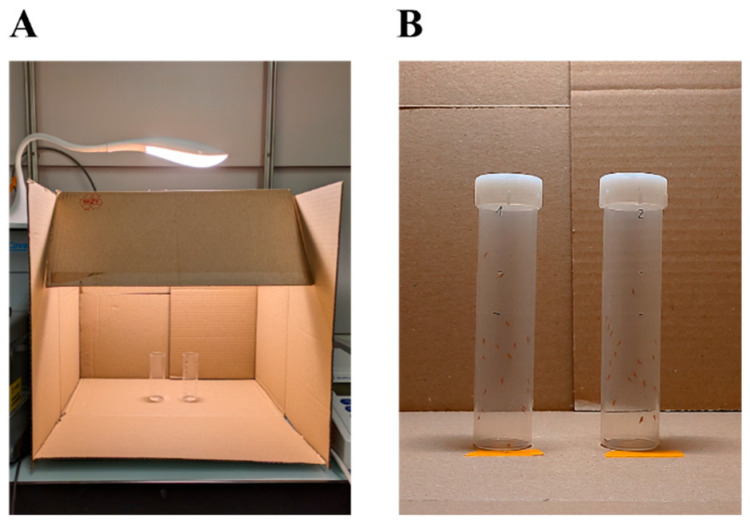
Negative geotaxis assay to assess climbing activity after UHDR and CONV irradiation. (**A**) Setup of the negative geotaxis assay, consisting of a cardboard box with a light source positioned above and test tubes placed inside the box below the light. (**B**) Representative image showing flies climbing the walls of the test tubes during the assay. The 6 cm and 8 cm heights are marked on the tubes.

Flies were subjected to 300 Gy of CONV and UHDR irradiation using 9 MeV electron beams. One and seven days after irradiation, the vials were cooled on ice for 4 min to temporarily immobilize the flies and facilitate their transfer into food-free screw cap tubes (Sarstedt) marked at heights of 6 cm and 8 cm. After transfer, the tubes were maintained at room temperature for 15 min to allow the flies to recover and regain mobility. The tubes were then placed inside a cardboard box with a bright light source positioned above them (see also Figure A2 for set-up). The flies were gently tapped to the bottom of the tube, and their climbing activity was recorded over a 10 s period. This process was repeated three times per vial. The percentage of flies that successfully crossed the 6 cm and 8 cm marks was measured to quantify their climbing performance.

### 2.5. Lipid Peroxidation Staining

Flies were subjected to CONV or single high-dose UHDR irradiation with doses of 300 Gy delivered in a single fraction using 9 MeV electron beams. Dissected fly brains were fixed in 4% paraformaldehyde for 20 min 1 h following irradiation, followed by three washes in PBS to remove residual fixative. Samples were then blocked for at least 1 h at room temperature in PBST (PBS with 0.2% Triton X-100) containing 10% HINGS. Whole brains were incubated with 4-Hydroxynonenal (4-HNE) polyclonal antibody and AbBy Fluor-488-conjugated (Bioss, Woburn, MA, USA USA) at a dilution of 1:100. After incubation, excess antibody was removed with PBS washes, and samples were mounted on standard microscope slides. Imaging was performed using a Leica SP8 laser confocal microscope at 10× magnification. Image analysis and area quantification were conducted with Imaris software version 10.2. The antibody-positive area was normalized to the total brain area to obtain the percentage of antibody coverage.

### 2.6. Statistical Analysis

Data analysis was conducted using GraphPad Prism v10.1.0. Lifespan curves were compared using the log-rank (Mantel–Cox) test. One-way ANOVA was performed for the negative geotaxis assay and lipid peroxidation measurements, and results were presented as the mean ± SEM. Differences were considered significant at *p* < 0.05.

## 3. Results

### 3.1. UHDR-RT of D. melanogaster with a Clinical Linear Accelerator

We assessed differential biological responses to UHDR and CONV irradiation in *D. melanogaster*, a well-established model system for genetic manipulation in preclinical research. Adult flies were subjected to UHDR and CONV (0.5 Gy/s) irradiation using a clinical linear accelerator with 16 MeV and 9 MeV electron beams (TrueBeam SN 1001, Varian Medical Systems), which was converted to deliver FLASH electron beams. A detailed description of the conversion has previously been published [17]. The irradiation setup is shown in [25].

### 3.2. Single High-Dose Delivery of UHDR Irradiation Is Less Toxic than CONV Irradiation to D. melanogaster Adult Flies

**Figure 1:**
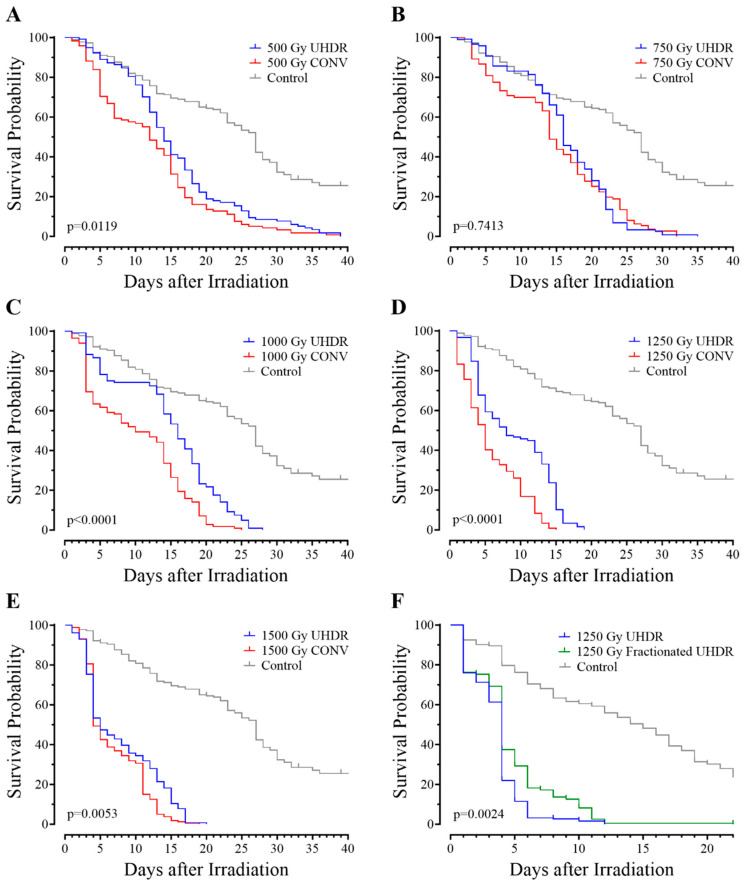
Single high-dose UHDR irradiation shows extended lifespan compared to CONV irradiation. Kaplan–Meier survival curves comparing 16 MeV electron UHDR and CONV irradiation using 500 Gy (**A**), 750 Gy (**B**), 1000 Gy (**C**), 1250 Gy (**D**), and 1500 Gy (**E**). (**F**) Kaplan–Meier survival curve comparing 1250 Gy of single high-dose and split-dose UHDR irradiation using 9 MeV electrons. Abbreviations: UHDR = ultra-high dose rate irradiation group; CONV = conventional irradiation group.

Lifespan serves as a highly effective endpoint for toxicity studies in *D. melanogaster*, given its relatively short life cycle of only 60–90 days which can vary depending on genetic background and maintenance conditions [26]. Initially, we assessed the effects of 16 MeV electron beams delivered as single high-dose UHDR or CONV irradiation on the lifespan of *D. melanogaster*. Pooled data from three independent lifespan assays for each dose, displayed as Kaplan–Meier survival curves, are shown in Figure 1A–E. Details of the individual lifespan assays are provided in Supplementary Figures S1 and S2. (See also Table 1 for detailed statistical informations.)

**Table 1:** Statistics for individual single high-dose UHDR and CONV irradiation lifespan assays.

Dose (Gy)	Exp.	*p*-Value (vs. UHDR)	Mean Lifespan (Days ± SEM)	Max. Lifespan (Days)	No. of Censored Flies	Total No. of Flies
500	UHDR 1		20.63 (±1.21)	39	0	40
	CONV 1	0.0008	12.68 (±1.40)	37	0	38
	UHDR 2		12.35 (±0.80)	24	1	38
	CONV 2	0.0100 *	15.25 (±1.10)	32	0	40
	UHDR 3		13.60 (±1.42)	39	0	40
	CONV 3	0.0346	9.00 (±1.20)	39	0	40
750	UHDR 1		19.05 (±0.88)	35	0	40
	CONV 1	0.0197	13.34 (±1.22)	29	2	40
	UHDR 2		17.51 (±0.92)	25	0	41
	CONV 2	0.0044 *	20.55 (±1.02)	32	0	40
	UHDR 3		11.59 (±0.95)	25	0	37
	CONV 3	0.0742	9.692 (±0.88)	25	1	40
1000	UHDR 1		18.15 (±0.63)	28	0	40
	CONV 1	0.0069	15.72 (±0.54)	20	0	36
	UHDR 2		18.65 (±0.86)	26	0	40
	CONV 2	<0.0001	12.47 (±0.94)	25	1	39
	UHDR 3		7.30 (±0.81)	16	0	40
	CONV 3	<0.0001	3.05 (±0.18)	6	0	40
1250	UHDR 1		7.87 (±0.78)	19	0	39
	CONV 1	0.2179	6.80 (±0.50)	14	0	39
	UHDR 2		11.46 (±0.79)	19	0	39
	CONV 2	<0.0001	8.73 (±0.63)	15	0	40
	UHDR 3		8.05 (±0.85)	16	0	40
	CONV 3	<0.0001	2.00 (±0.19)	5	0	40
1500	UHDR 1		7.00 (±1.01)	14	0	19
	CONV 1	0.0184	4.45 (±0.25)	7	0	20
	UHDR 2		5.14 (±0.48)	13	0	36
	CONV 2	0.0819	4.30 (±0.28)	9	0	40
	UHDR 3		8.87 (±0.57)	20	1	99
	CONV 3	0.0138	8.23 (±0.45)	19	0	100

* CONV results in a significantly longer lifespan compared to UHDR.

Significant lifespan extensions were observed in UHDR-irradiated groups compared to CONV-irradiated groups at doses of 500 Gy, 1000 Gy, 1250 Gy, and 1500 Gy, with the largest differences observed at 1000 Gy and 1250 Gy. At 500 Gy, the mean lifespan for UHDR-irradiated flies was 15.61 ± 0.76 days (mean lifetime ± SEM), higher than the 12.31 ± 0.75 days observed for CONV irradiation. At 1000 Gy, UHDR irradiation resulted in a mean lifespan of 14.70 ± 0.65 days, in contrast to 10.19 ± 0.62 days with CONV irradiation. At 1250 Gy, the UHDR-irradiated group showed a mean lifespan of 9.12 ± 0.49 days compared to 5.83 ± 0.38 days for CONV irradiation. Finally, at 1500 Gy, UHDR irradiation demonstrated a mean lifespan of 7.76 ± 0.42 days, exceeding the 6.78 ± 0.33 days seen with CONV irradiation. No significant difference in the overall lifespan was seen at 750 Gy despite an increased mean lifespan of 16.18 ± 0.60 days in UHDR-irradiated flies compared to 14.59 ± 0.73 days for CONV irradiation. These results demonstrate a reduced toxicity of UHDR irradiation on the lifespan of adult *D. melanogaster* flies.

Next, lifespan experiments were performed to compare split-dose UHDR, delivered in five fractions with 30 s intervals (split doses), and single high-dose UHDR. The split-dose UHDR regimen resulted in a modest but statistically significant increase in lifespan at 1250 Gy, with the mean lifespan rising from 3.57 ± 0.15 for single high-dose UHDR to 4.64 ± 0.23 for split-dose UHDR ( Figure 1F with pooled data from two independent lifespan assays). Details of each individual experiment are provided in Table 2. The Kaplan–Meier survival curves for each separate experiment are included in Supplementary Figure S3.

### 3.3. CONV Irradiation but Not UHDR Irradiation Induces Locomotor Deficits in D. melanogaster

**Figure 2:**
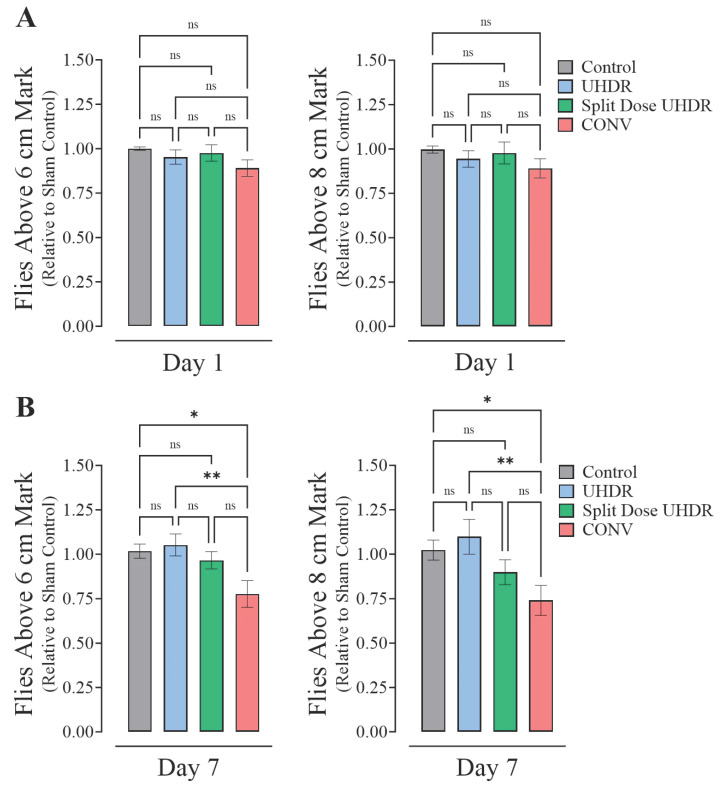
CONV irradiation, but not UHDR, leads to delayed locomotor deficits in *D. melanogaster*. (**A**) Proportion of flies that crossed the 6 cm (**left**) and 8 cm (**right**) marks after 10 s, measured on day one post-irradiation. (**B**) Proportion of flies that crossed the 6 cm (**left**) and 8 cm (**right**) marks after 10 s, measured on day seven post-irradiation. Data represent the mean and SEM, with a minimum of 179 flies per group. * *p* < 0.05; ** *p* < 0.01. Abbreviations: UHDR = ultra-high dose rate; CONV = conventional.

The negative geotaxis assay is a widely used method for assessing locomotor function in *D. melanogaster*. This behavioral assay measures the flies’ ability to climb against gravity, offering important insights into their motor skills and overall neurological health [27,28,29]. Thus, a longitudinal study was performed to assess the effects of single high-dose and split-dose UHDR, as well as CONV irradiation, on motor function in *D. melanogaster* using the negative geotaxis assay. To conduct the assay, flies were transferred into test tubes and placed in a box with a light source positioned above the box opening. The climbing ability of the flies was assessed one and seven days following irradiation with 300 Gy using 9 MeV electron beams. On day one post-irradiation, no significant differences in climbing ability were observed (Figure 2A). However, seven days after irradiation, a significantly higher proportion of flies in the single high-dose and unirradiated groups reached the 6 cm and 8 cm marks within 10 s compared to the CONV group (Figure 2B). Flies in the split-dose UHDR group exhibited a slight but non-significant reduction in climbing activity. These results demonstrate that UHDR irradiation, particularly when delivered as a single high-dose, preserves locomotor function in *D. melanogaster*, whereas CONV irradiation significantly impairs their climbing activity.

### 3.4. UHDR Irradiation Mitigates Radiation-Induced Ferroptosis Compared to CONV Irradiation in D. melanogaster

**Figure 3:**
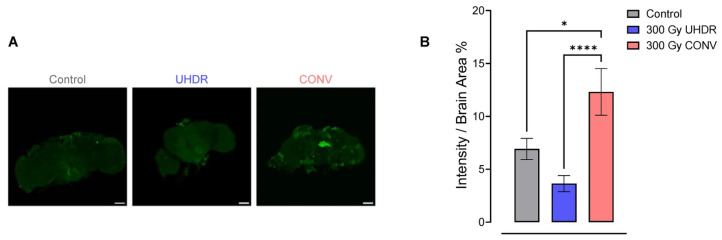
UHDR irradiation mitigates radiation-induced ferroptosis compared to CONV irradiation in *D. melanogaster*. (**A**) Representative 4-Hydroxynonenal staining images of *D. melanogaster* brains after UHDR and CONV irradiation with 300 Gy and (**B**) quantitative assessment (intensity/brain area). * *p* < 0.05; **** *p* < 0.0001.

Several mechanisms for the FLASH effect have been proposed using different preclinical animal models [15]. However, the exact way UHDR-RT reduces normal tissue damage remains unclear, underscoring the need for innovative models for mechanistic investigations. Ferroptosis, a regulated form of cell death driven by iron-dependent lipid peroxidation, has been implicated in the FLASH effect [30]. The reactive carbonyl species 4-Hydroxynonenal (4-HNE), a major product of lipid peroxidation, is widely used to assess lipid peroxidation in biological tissues [31]. In our study, 4-HNE staining revealed increased levels of lipid peroxidation in *D. melanogaster* brains following CONV irradiation with 300 Gy, whereas this effect was not observed after single high-dose UHDR irradiation (Figure 3).

## 4. Discussion

In this study, we establish adult *D. melanogaster* flies as an effective model for investigating both early and late irradiation-induced toxicities of UHDR and CONV-RT by assessing lifespan and locomotor function. Significant lifespan extensions were observed with UHDR irradiation at doses of 500, 1000, 1250, and 1500 Gy compared to CONV irradiation, with the most substantial benefits occurring at 1000 and 1250 Gy, suggesting an optimal dose range for observing the FLASH effect in adult *D. melanogaster* flies. Intriguingly, the incorporation of four intra-fraction pauses of 30 s (split dose) further enhanced lifespan compared to single high-dose UHDR irradiation. In the negative geotaxis assay, we observed that single high-dose UHDR irradiation prevented locomotor dysfunction post-irradiation. Such behavioral assays provide valuable insight into the temporal dynamics of irradiation-induced toxicities, enabling comprehensive monitoring of functional outcomes in *D. melanogaster* over time.

### 4.1. UHDR Irradiation and D. melanogaster

Adult *D. melanogaster* flies present a novel and powerful whole-body model system for preclinical research into UHDR irradiation. The structural and physiological similarities of their tissues and organs to those of mammals, coupled with an advanced genetic toolkit enabling precise genetic manipulation, make *D. melanogaster* an optimal system for investigating the differential radiobiological effects of UHDR and CONV irradiation. Thus, this model organism can contribute to both mechanistic studies and the identification of optimal beam characteristics and irradiation protocols. Given the variability observed across the multiple UHDR-RT studies, it is important to identify the precise physical beam irradiation parameters to reliably induce the differential biological responses to UHDR-RT and CONV-RT. These parameters extend beyond the average dose rate and encompass factors such as total dose, pulse frequency, pulse duration, dose per pulse, and overall delivery time [32]. The lifespan of *D. melanogaster* offers a straightforward, high-throughput endpoint for assessing these parameters with substantial statistical power. We observed substantial variability in the lifespan of both irradiated and control flies across different experiments (see Supplementary Figures S1 and S2). These differences may have been influenced by variations in environmental conditions, food, and larval crowding, all of which are known to affect fitness-related traits, including lifespan. Additionally, stress during public transport (15 min) from the fly incubator to the irradiations facility may have impacted the flies, particularly in colder weather [33,34]. Despite variability between control groups and experiments, UHDR consistently demonstrated improved survival compared to CONV irradiation across several independent experiments. Pooling the lifespan data helped to mitigate the effects of potential confounding factors, such as food contamination or environmental stressors, that may have influenced individual experiments.

While these physical parameters are crucial for clinical translation, the underlying biological mechanisms mediating the FLASH effect also demand exploration to fully harness its therapeutic potential. For example, ferroptosis, a regulated form of cell death driven by iron-dependent lipid peroxidation, has been implicated in the FLASH effect. We here identified that UHDR irradiation mitigates radiation-induced lipid peroxidation in *D. melanogaster* further supporting that reduced amounts of ferroptosis as a mode of cell death contributes to reduced normal tissue toxicity in response to UHDR in comparison to CONV irradiation [30].

As such, the genetic toolbox offered by *D. melanogaster* and the simple administration of pharmacological compounds could be of pivotal help to gain further mechanistic insights. High-throughput experimentation, low maintenance costs, short generation times, and exemption from ethical approval requirements will further enhance its potential to drive significant advancements in UHDR-RT research, particularly in the clinical setting.

At the time of our investigation, only one study has so far explored UHDR in *D. melanogaster* [23], albeit using X-rays, which will not be available for clinical use in the near future [35,36]. In contrast to working with adult flies, Hart and colleagues used the less robust *D. melanogaster* larvae system in their study to compare the effects of CONV irradiation (0.2–0.4 Gy/s) to UHDR irradiation (210 Gy/s). Their results showed that larvae irradiated with 24 Gy exhibited a 68% higher eclosion rate with UHDR compared to CONV irradiation. Additionally, UHDR irradiations with 22 Gy resulted in significantly increased survival probabilities and extended median survival in comparison to CONV irradiations. These findings align with our own, further supporting the superior survival outcomes associated with UHDR irradiation.

### 4.2. Split-Dose UHDR Irradiation in D. melanogaster

Classic radiobiological aspects, such as tumor hypoxia and dose fractionation, remain to be addressed prior to successful clinical translation of UHDR-RT. However, these issues are complex and remain unresolved at this stage. This is demonstrated in our experiments, where split-dose UHDR irradiation resulted in improved survival outcomes, but comparatively diminished climbing activity compared to single high-dose UHDR. This discrepancy may be attributed to distinct toxicity mechanisms impacting survival and locomotor function. Split-dose UHDR over time resulted in a reduction in intermediate and long-term toxicity. A potential radiobiological explanation for the improved lifespan observed with split-dose UHDR irradiation in comparison with single high-dose UHDR irradiation, may involve rapid cellular adaptation mechanisms taking place after the delivery of the first 250 Gy dose. During the pause in between the split doses, cells subjected to UHDR irradiation might activate protective mechanisms and mitigate the radiation-induced damage. These adaptive responses likely differ from those characterizing conventionally fractionated RT regimens, suggesting unique radiobiological processes at play in UHDR irradiation. The lower dose per splitted dose of 60 Gy within the negative geotaxis assay might fall below the threshold required to activate such protective mechanisms. Eventually, dissecting the molecular processes in response to these high doses applied with UHDRs will be highly informative to understand UHDR physical chemistry. Alternatively, the differential effect of split-dose UHDR irradiation on lifespan and climbing activity might also reflect variations in irradiation parameters, such as average dose rate and dose per pulse, between the two experimental setups.

In summary, the adult *D. melanogaster* fly presents a powerful and versatile whole-body model system for studying UHDR-RT. Its tissues and organs share significant structural and physiological similarities with those of mammals, while its advanced genetic tools allow precise manipulation, making it suitable for exploring the distinct radiobiological effects of UHDR and conventional irradiation. We recognize that the higher total doses are not comparable to patient treatment and that only whole-body RT can be performed with *D. melanogaster*. The practical advantages of the model—such as low maintenance costs, short lifetime, potential for high-throughput experiments and exemption from ethical approvals in many countries—outweigh these limitations for gaining insights into the biological processes induced by UHDR-RT. Furthermore, *D. melanogaster* tumor models also exist [37], which can be used to directly determine a FLASH effect in this versatile animal model. This combination of features enables *D. melanogaster* to drive both mechanistic studies and the optimization of beam characteristics and irradiation protocols. Ultimately, understanding the key parameters for reliably triggering the FLASH effect is essential for establishing UHDR-RT as a groundbreaking radiation therapy approach in cancer treatment. As a result, UHDR-RT has the potential to significantly expand the therapeutic options for various types of cancer.

## 5. Conclusions

In summary, adult *D. melanogaster* is a powerful whole-body model for studying UHDR-RT, offering structural and physiological similarities in its tissues and organs to those of mammals, advanced genetic tools, and practical advantages such as low cost, short lifespan, and high experimental throughput. While higher total doses and whole-body irradiation limit direct clinical comparability, these are outweighed by the model’s utility for investigating UHDR-RT radiobiology. Existing *D. melanogaster* tumor models further enable direct assessment of the FLASH effect [38]. Genetic studies with *D. melanogaster* can now be used to elucidate underlying MoAs, which might be related to a differential redox biology induced by UHDR-RT in comparison to CONV RT. Thereby the full potential of UHDR-RT and its benefits in the clinic could be even further improved. Together, these features make *D. melanogaster* suitable for mechanistic studies and optimization of irradiation protocols, ultimately advancing UHDR-RT as a transformative cancer therapy.

## Figures and Tables

**Table 2:** Statistics for individual single high-dose and split-dose UHDR irradiation lifespan assays.

Dose (Gy)	Exp.	*p*-Value (vs. UHDR)	Mean Lifespan (Days ± SEM)	Max. Lifespan (Days)	No. of Censored Flies	Total No. of Flies
1250	Single–High 1		2.14 (±0.18)	10.00	0	79
	Split Dose 1	0.0358	2.77 (±0.28)	10.00	0	79
	Single–High 2		4.57 (±0.16)	12.00	0	112
	Split dose 2	0.0001	5.89 (±0.29)	22.00	0	119

## Data Availability

Research data are stored in an institutional repository and will be shared upon request to the corresponding author.

## Supplementary Materials

The following supporting information can be downloaded at https://www.mdpi.com/article/10.3390/antiox15060736/s1: Figure S1: Individual lifespan experiments comparing single high-dose UHDR-RT and CONV-RT; Figure S2: Individual lifespan experiments comparing single high-dose UHDR-RT and CONV-RT; Figure S3: Individual lifespan experiments comparing single high-dose and split-dose UHDR; Table S1: Breakdown of the DPP, average and instantaneous dose rates for different irradiation setups. The reported values correspond to the average (minimum–maximum) among the passive detectors: EBT3, HD-V2 and myOSLchip; Table S2: Beam parameters according to the reporting recommendations.

## Abbreviations

The following abbreviations are used in this manuscript:

UHDR	ultra-high dose rates
CONV	conventional
G	Gy
RT	Radiotherapy
D	Drosophila
