# Supplementary material for: Deciphering Ultra-High Dose Rate Irradiation with Drosophila melanogaster
Source: Antioxidants (Basel). 2026 Jun 10;15(6):736. doi: 10.3390/antiox15060736 (PMC13296059; doi:10.3390/antiox15060736)
Supplement: Supplementary file 1 [file antioxidants-15-00736-s001.zip › 1 List of the Supplementary Materials.pdf]

Supplementary Figure S1: Individual lifespan experiments comparing single high-dose UHDR-RT and CONV-RT. Kaplan-Meier survival curves illustrating lifespan outcomes for individual experiments involving 500 Gy (A-C), 750 Gy (D-F) of single high-dose UHDR and CONV irradiation, delivered using 16 MeV electrons. *Abbreviations*: UHDR = ultra-high dose rate; CONV = conventional.

Supplementary Figure S2: Individual lifespan experiments comparing single high-dose UHDR-RT and CONV-RT. Kaplan-Meier survival curves illustrating lifespan outcomes for individual experiments involving 1000 Gy (A-C), 1250 Gy (D-F), 1500 Gy (G-I) of single high-dose UHDR and CONV irradiation, delivered using 16 MeV electrons. *Abbreviations*: UHDR = ultra-high dose rate; CONV = conventional.

Supplementary Figure S3: Individual lifespan experiments comparing single high-dose and split dose UHDR. Kaplan-Meier curves illustrating lifespan outcomes for individual experiments involving 1250 Gy of single high-dose and split dose UHDR, delivered using 9 MeV electrons. (A) Lifespan data comparing Single-High 1 and Split dose 1. (B) Lifespan data comparing Single-High 2 and Split dose 2. *Abbreviations*: UHDR = ultra-high dose rate.

Supplementary Table S1. Breakdown of the DPP, average and instantaneous dose rates for different irradiation setups. The reported values correspond to the average (minimum - maximum) among the passive detectors: EBT3, HD-V2 and myOSLchip.

Supplementary Table S2. Beam parameters according to the reporting recommendations.
