# Supplementary material for: Deciphering Ultra-High Dose Rate Irradiation with Drosophila melanogaster
Source: Antioxidants (Basel). 2026 Jun 10;15(6):736. doi: 10.3390/antiox15060736 (PMC13296059; doi:10.3390/antiox15060736)
Supplement: Supplementary file 1 [file antioxidants-15-00736-s001.zip › 2 List of Supplementary Materials incl. Figures and Tables.pdf]

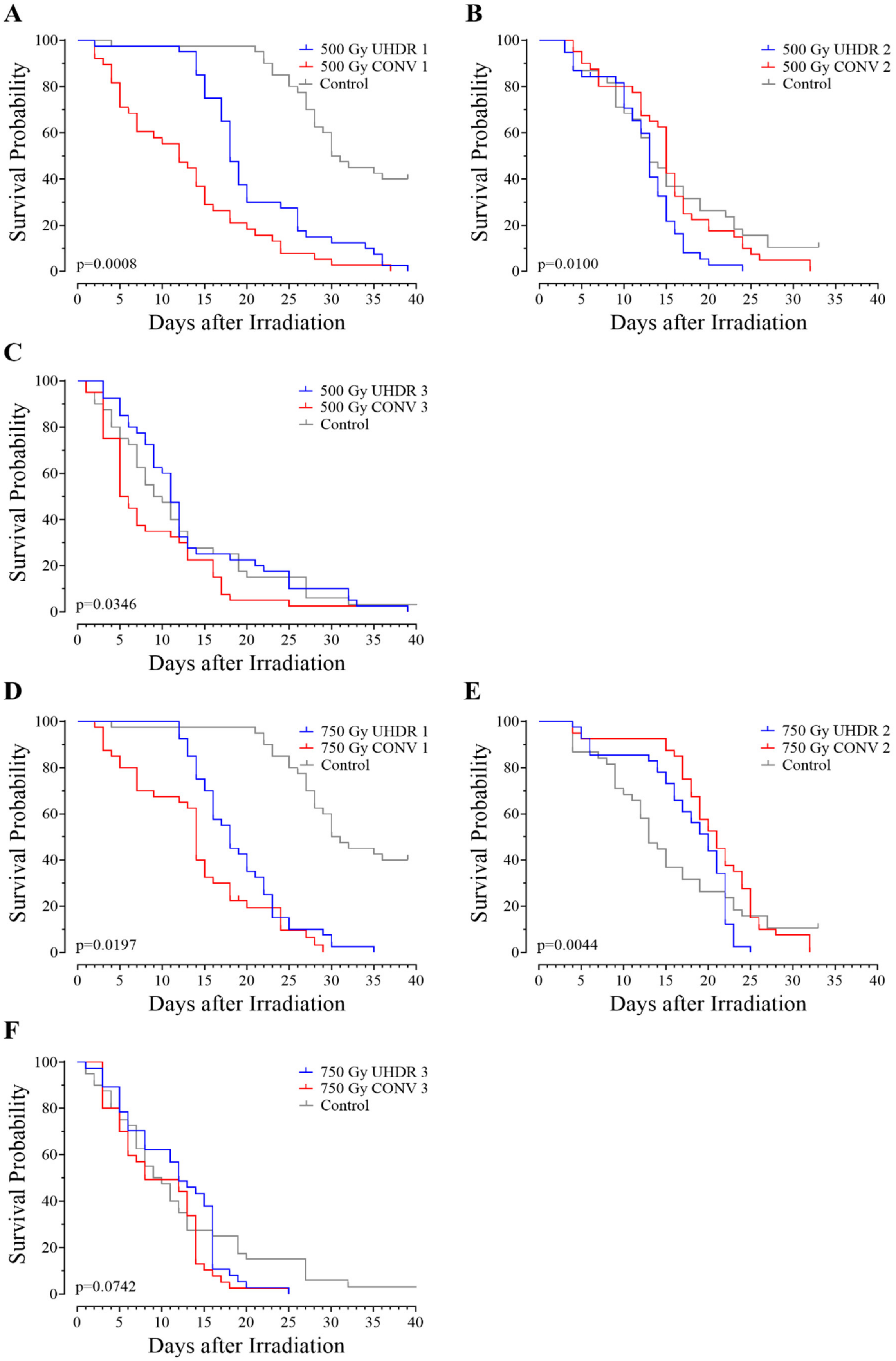
A
Survival Probability
Days after Irradiation
500 Gy UHDR 1
500 Gy CONV 1
Control
p=0.0008
B
Survival Probability
Days after Irradiation
500 Gy UHDR 2
500 Gy CONV 2
Control
p=0.0100
C
Survival Probability
Days after Irradiation
500 Gy UHDR 3
500 Gy CONV 3
Control
p=0.0346
D
Survival Probability
Days after Irradiation
750 Gy UHDR 1
750 Gy CONV 1
Control
p=0.0197
E
Survival Probability
Days after Irradiation
750 Gy UHDR 2
750 Gy CONV 2
Control
p=0.0044
F
Survival Probability
Days after Irradiation
750 Gy UHDR 3
750 Gy CONV 3
Control
p=0.0742

Supplementary Figure S1: Individual lifespan experiments comparing single high-dose UHDR-RT and CONV-RT. Kaplan-Meier survival curves illustrating lifespan outcomes for individual experiments involving 500 Gy (A-C), 750 Gy (D-F) of single high-dose UHDR and CONV irradiation, delivered using 16 MeV electrons. *Abbreviations*: UHDR = ultra-high dose rate; CONV = conventional.

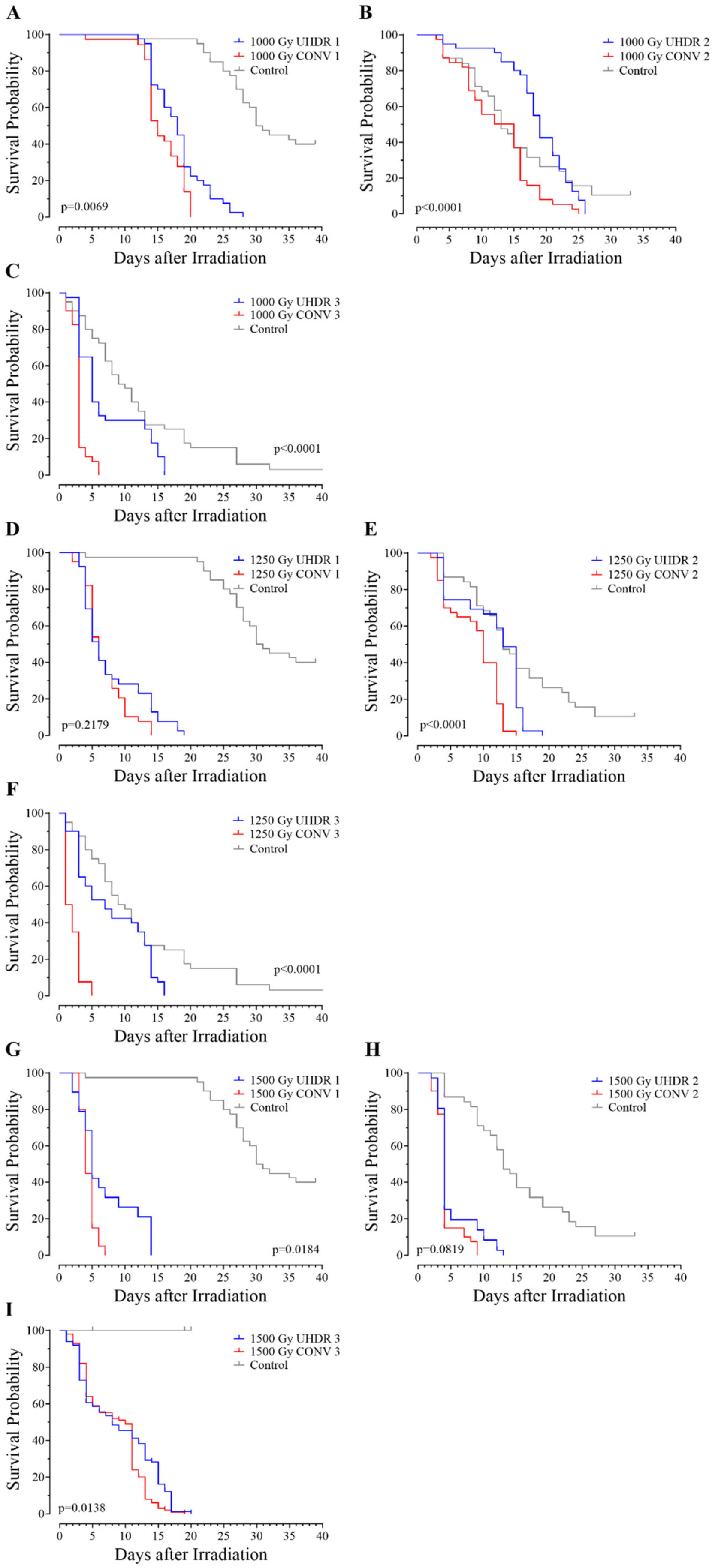
A
1000 Gy UHDR 1
1000 Gy CONV 1
Control
p=0.0069
B
1000 Gy UHDR 2
1000 Gy CONV 2
Control
p<0.0001
C
1000 Gy UHDR 3
1000 Gy CONV 3
Control
p<0.0001
D
1250 Gy UHDR 1
1250 Gy CONV 1
Control
p=0.2179
E
1250 Gy UHDR 2
1250 Gy CONV 2
Control
p<0.0001
F
1250 Gy UHDR 3
1250 Gy CONV 3
Control
p<0.0001
G
1500 Gy UHDR 1
1500 Gy CONV 1
Control
p=0.0184
H
1500 Gy UHDR 2
1500 Gy CONV 2
Control
p=0.0819
I
1500 Gy UHDR 3
1500 Gy CONV 3
Control
p=0.0138
Survival Probability
Days after Irradiation

Supplementary Figure S2: Individual lifespan experiments comparing single high-dose UHDR-RT and CONV-RT. Kaplan-Meier survival curves illustrating lifespan outcomes for individual experiments involving 1000 Gy (A-C), 1250 Gy (D-F), 1500 Gy (G-I) of single high-dose UHDR and CONV irradiation, delivered using 16 MeV electrons. *Abbreviations*: UHDR = ultra-high dose rate; CONV = conventional.

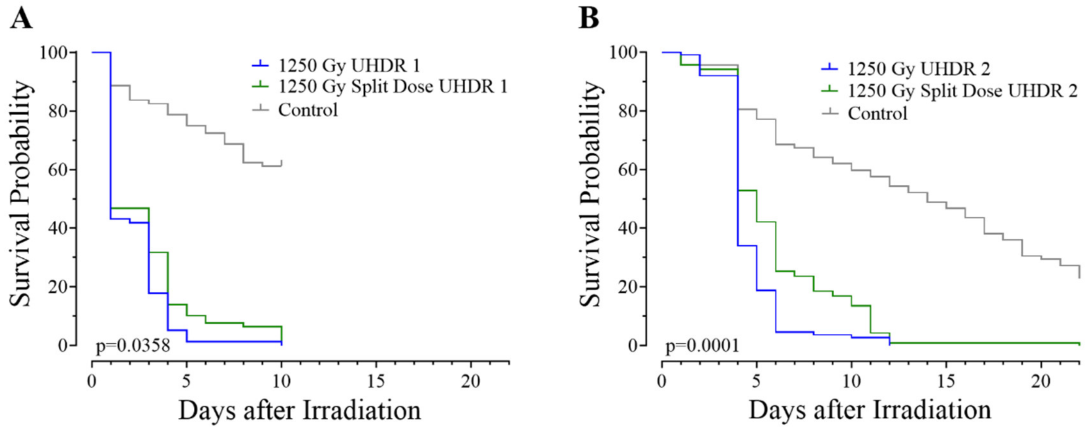

Supplementary Figure S3: Individual lifespan experiments comparing single high-dose and split dose UHDR. Kaplan-Meier curves illustrating lifespan outcomes for individual experiments involving 1250 Gy of single high-dose and split dose UHDR, delivered using 9 MeV electrons. (A) Lifespan data comparing Single-High 1 and Split dose 1. (B) Lifespan data comparing Single-High 2 and Split dose 2. *Abbreviations*: UHDR = ultra-high dose rate.

SSD [cm]	Linac modality	Dose per pulse [Gy]	Instantaneous dose rate [Gy/s]	Average dose rate [Gy/s]
60	CONV	$2.3 \cdot 10^{-3}$ ($2.0 \cdot 10^{-3}$ - $2.4 \cdot 10^{-3}$)	$5.0 \cdot 10^{2}$ ($4.4 \cdot 10^{2}$ - $5.3 \cdot 10^{2}$)	0.45 (0.40 - 0.47)
60	UHDR	3.19 (2.99 - 3.28)	$7.1 \cdot 10^{5}$ ($6.6 \cdot 10^{5}$ - $7.3 \cdot 10^{5}$)	638 (597 - 657)
40	UHDR	16.4 (15.8 - 17.0)	$3.7 \cdot 10^{6}$ ($3.5 \cdot 10^{6}$ - $3.8 \cdot 10^{6}$)	3290 (3150 - 3410)

Supplementary Table S1. Breakdown of the DPP, average and instantaneous dose rates for different irradiation setups. The reported values correspond to the average (minimum - maximum) among the passive detectors: EBT3, HD-V2 and myOSLchip.

Quantity	Abbreviation	Values
**Minimal reporting**		
General description		
Device name	FLEX estension for a TrueBeam v2.7.5	
Accelerator type	Electron linac	
Dose delivery technique	Scattering and collimation	
Traceability and dosimetry code of practice used	Detectors cross calibrated at conventional dose rate to a PTW Roos chamber, which was calibrated in the Swiss primary standard laboratory METAS. Reference dosimetry performed according to SSRMP recommendation nr. 10.	
Additional key information about delivery	None	
Preclinical: Biological system(s), model(s), endpoint(s)<br><br>Clinical: Site, diagnosis, stage, cohort characteristics	Yellow-white Drosophila Melanogster. Multiple endpoints.	
Additional key information (including imaging)<br><br>about irradiated systems/models/patients	The animals are contained in a cylindrical vial with food at the bottom and a cap at the top. The cap at the top allows air to flow freely but blocks the animals from escaping the vial. The animals live in an atmosphere that is equivalent to air. For the irradiation the animals are gently pushed down towards the food and the cap lowered such that they are confined in a space <5 mm along the beam direction (vertical dimension). In the plane orthogonal to the beam direction the animals are confined in a circular space of 20-25 mm diameter. Between 1h and 4h after the irradiation the animals are temporarily anaesthetised with CO_2, the cap is lifted, and they are again free to move in a vertical space of > 2 cm.	
Non-temporal beam parameters		
Radiation type and nominal beam energy	E	16 MeV and 9 MeV electrons
Beam dose at reference point or volume	D_{beam}	Multiple doses used. See main manuscript
Reference point or volume specification	P_{ref} or V_{ref}	Full uniform body dose to the Drosophila. Dose specified at the

		surface of the food, which corresponds to the location where the animals are confined during the irradiations.
Source-to-surface distance	SSD	Multiple SSD used. See main manuscript
Field size	FS	Multiple field sizes used. See main manuscript
Temporal beam structure parameters		
Pause before next beam	*ΔT*beam	At least 7.5 s
Beam-on time	*T*beam	(#pulse – 1) x 5 ms
Number of pulses for beam	#pulse	Multiple pulses number used. See main manuscript.
Pulse length§	*t*pulse	4.5 μs
Pulse repetition frequency	*PRF*	200 Hz
Pulse charge	Qpulse	Approx 500 nC measured by inserting the photon bremsstrahlung target along the beam
Number of bunches per pulse	#bunch	-
Bunch length	*t*bunch	-

Bunch repetition frequency	*BRF*	-
Bunch charge	Qbunch	-
**Optimal reporting**		
Derived and additional parameters		
Average dose rate at reference point	ADR	Multiple values. Supplementary table above.<br><br>Calculated with: Dbeam / Tbeam
Instantaneous dose rate at reference point	IDR	Multiple values. Supplementary table above.<br><br>Calculated with: *DPP* / $t$pulse
Dose per pulse	DPP	Multiple values. Supplementary table above.
Representative 2D dose distribution of beam, PDD and lateral profiles	-	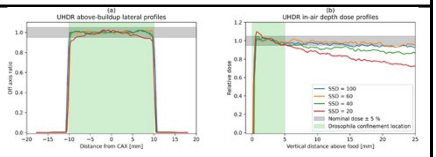
ADR-volume histograms of beam for relevant structures	-	-

Supplementary Table S2. Beam parameters according to the reporting recommendations.
